# Patients' assessment of care for type 2 diabetes: Results of the Patient Assessment of Chronic Illness Care scale in a Danish population

**DOI:** 10.1186/s12913-021-07051-6

**Published:** 2021-10-09

**Authors:** Anne Frølich, Ann Nielsen, Charlotte Glümer, Christian U Eriksen, Helle Terkildsen Maindal, Bodil Helbech Kleist, Hanne Birke, Anders Stockmarr

**Affiliations:** 1 Section of General Practice, Department of Public Health, University of Copenhagen, 1356 K, Copenhagen, Denmark; 2 Innovation and Research Centre for Multimorbidity, Slagelse Hospital, 4200 Slagelse, Denmark; 3 Danish Dementia Research Centre, Rigshospitalet, University of Copenhagen, 2100 Copenhagen, Denmark; 4 Center for Diabetes, KBH V, 1620 Copenhagen, Denmark; 5 Danish Health Authority, Copenhagen, Denmark; 6 Department of Public Health, Aarhus University, Aarhus, Denmark; 7 Local Government Denmark (KL), Copenhagen, Denmark; 8 Center for clinical Research and Prevention, Frederiksberg Hospital, Frederiksberg, Denmark; 9 Department of Applied Mathematics and Computer Science, Technical University of Denmark, 2800 Kgs. Lyngby, Denmark

**Keywords:** PACIC, Validation, Diabetes Mellitus, Type 2, Patient-Centred Care

## Abstract

**Background:**

The Patient Assessment of Chronic Illness Care (PACIC) scale is the most appropriate for assessing self-reported experience in chronic care. We aimed to validate the PACIC questionnaire by (1) assess patients’ perception of the quality of care for Danish patients with type 2 diabetes, (2) identify which factors are most important to the quality of care designated by the five subscales in PACIC, and (3) the validity of the questionnaire.

**Methods:**

A survey of 7,745 individuals randomly selected from the National Diabetes Registry. Descriptive statistics inter-item and item-rest correlations and factor analysis assessed the PACIC properties. Quality of care was analysed with descriptive statistics; linear and multiple regression assessed the effect of forty-nine covariates on total and subscale scores.

**Results:**

In total, 2,696 individuals with type 2 diabetes completed ≥ 50 % of items. The floor effect for individual items was 8.5–74.5 %; the ceiling effect was 4.1–47.8 %. Cronbach’s alpha was 0.73–0.86 for the five subscales. The comparative fit index (CFI) and the Tucker–Lewis index (TLI) were 0,87, and 0,84, respectively. Mean PACIC score was 2.44 (± 0.04). Respondents, who receive diabetes care primarily at general practice and outpatient clinics had higher scores compared to those receiving care at a private specialist. Receiving rehabilitation was followed by higher scores in all subscales. Those 70 years or older had lower mean total and subscale scores compared to younger patient groups.

A higher number of diabetes visits were associated with higher total scores; a higher number of emergency department visits were associated with lower total scores. The effects of healthcare utilisation on subscale scores varied.

**Conclusions:**

These results provide insight into variations in the quality of provided care and can be used for targeting initiatives towards improving diabetes care. Factors important to the quality of perceived care are having a GP or hospital outpatient clinic as the primary organization. Also having a higher number of visits to the two organizations are perceived as higher quality of care as well as participating in a rehabilitation program. Floor and ceiling effects were comparable to an evaluation of the PACIC questionnaire in a Danish population. Yet, floor effects suggest a need for further evaluation and possible improvement of the PACIC questionnaire in a Danish setting. Total PACIC scores were lower than in other healthcare systems, possible being a result of different contexts and cultures, and of a need for improving diabetes care in Denmark.

**Supplementary Information:**

The online version contains supplementary material available at 10.1186/s12913-021-07051-6.

## Background

Disease management programs for type 2 diabetes patients have been implemented in the Capital Region of Denmark to support evidence-based care. The programs are founded based on the Chronic Care Model (CCM) that is one approach to improving chronic illness care [1]. Programs cover diagnosis, treatment, rehabilitation, and follow-up. The effectiveness of diabetes care increases by improving patient activation, information and self-management associated with behaviours, such as treatment adherence, a healthier diet and physical activity [2–4].

The twenty-item Patient Assessment of Chronic Illness Care (PACIC) questionnaire considers whether patients receive patient-centred care in alignment with the CCM. The PACIC is grouped into five subscales representing five care components of the CCM that is central to quality of care [5, 6]. The five care components are: patient activation, delivery system design/decision support, goal setting/tailoring care, problem-solving/contextual counselling, and follow-up/coordination [1, 7]. The PACIC has been translated into Danish and applied in a primary care setting in Denmark to assess data quality, internal consistency, and factorial structure [8]. The psychometric properties in that study were satisfactory, apart from floor and ceiling effects. Also, the subscales showed good model fit and could be used for separate sum scores. Later, the PACIC was applied in another Danish diabetes population to assess perceptions of care among asymptomatic diabetes individuals identified by a screening program and receiving intensive multifactorial treatment. Although the individuals were early in their disease trajectory, and thus the effect of treatment could exceed the disease burden, the intensive diabetes care did not worsen the perceptions of care compared to individuals receiving routine diabetes care[7]. Yet the overall low PACIC score indicated room for improvement in diabetes care [7]. Conversely, a German study comparing patients receiving usual care to those enrolled in a diabetes disease management program found that patients receiving structured evidence-based care scored significantly higher on all PACIC subscales [9]. A Dutch study reviewing 37 instruments to measure self-reported experience in chronic care concluded PACIC to be the most appropriate [10]. Our objective of the study was to (1) assess the quality of care for Danish patients with type 2 diabetes, and (2) identify which factors that are most important to perceived quality of care designated by the five subscales in PACIC, and (3) the validity of the questionnaire.

## Methods

### Population

In 2013, we randomly selected 8,000 individuals aged 40 years or older with diabetes living in the Capital Region from the National Diabetes Registry [11]. Of these, 255 had died or moved out of the region, and the PACIC was subsequently mailed to 7.745 individuals, along with a link to an identical electronic version. Three weeks later, we sent a reminder to non-respondents. In total, 4,190 patients completed the PACIC, yielding a response rate of 54.1 %. The inclusion criteria were persons 40 years or older with type 2 diabetes, living in the Capital region. The exclusion criteria were persons that had not diabetes and persons with type 1 diabetes. Of the responders, 2,810 had type 2 diabetes, while 1,380 patients (20 %) were excluded.

### PACIC scoring and additional variables

Each item was scored on a five-point scale ranging from one (no/never) to five (yes/always). Each subscale was scored by averaging scores for completed items, and the total score was calculated by averaging scores across all twenty items. In the Danish version, the wording of a few items was updated in collaboration with Judith Schaefer, developer of the PACIC [8]. The Danish version also includes items about diabetes type, the primary location of diabetes care and rehabilitation, and type(s) of received rehabilitation: diabetes education, smoking cessation, physical training, and dietician counselling. The Danish version can be provided by request to the authors.

PACIC scores were linked to national registers and health registers to obtain information on sex, age, education, occupation, cohabitation status, socioeconomic area, multimorbidity status, number of visits with a general practitioner (GP), annual diabetes control visits, planned and acute hospital admissions and bed days, and readmissions. The socioeconomic area was identified by proximity to one of four hospital planning areas that differ socioeconomically. Chronic conditions included allergy, depression, stroke, dementia, chronic heart disease, hypertension, chronic pulmonary disease, high cholesterol, cancer, arthritis, osteoporosis, chronic back pain, and schizophrenia. Comorbidity status was based on algorithms as described in Robinson [12]. Multimorbidity was defined as the presence of two or more chronic conditions.

### Statistical analyses

Of all 4,190 respondents, 1,380 (32.9 %) did not have diabetes or had type 1 diabetes and were excluded. Of the remaining 2,810 respondents, 114 respondents completed fewer than ten items and were excluded Thus, 2,696 respondents, who completed ten items or more, were included in the analysis. We used the expectation-maximisation (EM) algorithm to impute item values for 259 respondents who answered 50 % or more but less than 100 % of the items [13]. Comparative analyses without imputation were carried out.

Data quality was evaluated with standard summary statistics. Floor and ceiling effects were presented and analysed. Internal consistency was evaluated with correlational analyses within each subscale and between the five subscales using Cronbach’s alpha.

Additionally, confirmatory factor analysis was carried out, investigating the fit of a latent class for each of the 5 PACIC subscores to data. This factor structure was assessed by the model χ^2^ test, the Root Mean Square Error of Approximation (RMSEA), the Standardized Root Mean Square Residual (SRMR), the Tucker-Lewis Index (TLI) and the Comparative Fit Index (CFI). We considered a value of Cronbach’s alpha of 0.7 or above as satisfactory [14]. Descriptive analyses included means, standard deviations, floor and ceiling effects for the total score, for each of the five subscales, and means and standard deviations for the twenty individual items.

To identify factors affecting total PACIC scores, we used linear regression analysis; the nature of the PACIC score as an average of twenty scores across five subscales invites continuous modelling. Multiple regression analysis was used to examine the total PACIC score and each subscale score for relationships with the variables listed above, a total of forty-nine different factors. The factors education, occupation, and socioeconomic area were multi-level factors, while the remaining factors were binary or numeric. We used the following formula for modelling:

1 $$Y_i=\alpha_{education\left(i\right)}+\alpha_{occupation\left(i\right)}+\alpha_{area\left(i\right)}+\sum_{j=1}^{46}\beta_jX_{ij}+\epsilon_i,i=1,\dots n.$$

In formula (1), ${Y}_{i}$ denotes either the total PACIC score or one of the five subscale scores, while ${X}_{ij}$ denotes the value of the *j*^*th*^ of the forty-six binary or numeric factors for the *i*^th^ patient. The model was applied separately for the total PACIC score and each of the five subscale scores. The model reduction was done with the Likelihood Ratio method with a significance level of 5 %. Subsequently, a forward selection procedure was applied to detect statistical significances obscured by the top-down model reduction. The forward selection procedure did not result in any alteration of the top-down reduced models. Analyses were performed with the software R, version 3.1.0 [15].

## Results

### Respondent characteristics

Of the 2,810 respondents, 59.1 % were men, 78.4 % were older than 60 years, 65.4 % were retired, 80.7 % of respondents had no education or education of short duration. Most (70.5 %) received diabetes care from their GP, one fifth (21.8 %) received care at an outpatient clinic, and a minority of 0.4 % received care from a private specialist (Table 1).

**Table 1:** Characteristics of 2,810 respondents with type 2 diabetes

Sex		N (%)
Men		1,662 (59.1)
Women		1,148 (40.9)
Age in years		
	40–49	135 (4.8)
	50–59	475 (16.9)
	60–69	1,064 (37.9)
	70–79	811 (28.0)
	≥ 80	325 (11.6)
Education level (total years of education)		
	None (≤ 10)	890 (31.7)
	Short (11–14)	1,378 (49.0)
	Medium (15–16)	299 (10.6)
	Long (≥ 17)	160 (5.7)
	Not reported	83 (3.0)
Occupation		
	Working or in school	827 (29.4)
	Unemployed	30 (1.1)
	Long-term disability	72 (2.6)
	Early retirement	237 (8.4)
	Retirement	1,603 (57.0)
	Not reported	41 (1.5)
Cohabitation status		
	Living alone	1,099 (39.1)
	Living with spouse	1,711 (60.9)
Primary location of diabetes care		
	General practitioner	1,980 (70.5)
	Outpatient clinic	612 (21.8)
	Private specialist	12 (0.4)
	Not reported	49 (1.7)

### Quality of PACIC data

In total, 2,434 respondents (86.7 %) answered all twenty questions. Only 74 respondents (2.6 %) did not answer any of the questions. Response rates were similar for all items, and the distributions of answered and unanswered questions were similar for the five subscales. The rate of answered questions decreased with increasing age, and women had a lower rate of answered questions than men.

The floor effect was 8.5–74.5 %, with nineteen items higher than 15 %. The ceiling effect was 4.1–47.8 %, with eleven items of more than 15 %. Cronbach’s alpha varied between 0.73 and 0.86 for the 5 subscales. The mean inter-item correlation in the five subscales varied between 0.45 and 0.60. The inter-item correlation range was 0.34–0.73, and the item-rest correlation range was 0.47–0.79. Confirmatory factor analysis showed factor loadings for the twenty items ranging from 0.51 to 0.72. (Table 2)

**Table 2:** Results of the confirmatory factor analysis showing the standardized factor loadings and residuals for each item when modelled with its own scale

Over the past 12 months, when I received care for my chronic conditions, I was:	Standardized factor loading	Residual variance
**Patient activation** Item 1 Item 2 Item 3	0.629 0.634 0.626	0.027 0.027 0.027
**Delivery system design/decision support** Item 4 Item 5 Item 6	0.520 0.560 0.613	0.027 0.027 0.027
**Goal setting/tailoring** Item 7 Item 8 Item 9 Item 10 Item 11	0.666 0.692 0.510 0.517 0.623	0.025 0.025 0.025 0.025 0.025
**Problem solving/contextual** Item 12 Item 13 Item 14 Item 15	0.613 0.697 0.724 0.690	0.025 0.025 0.025 0.025
**Follow-up/coordination** Item 16 Item 17 Item 18 Item 19 Item 20	0.523 0.564 0.571 0.586 0.555	0.025 0.025 0.025 0.025 0.025

The factor structure assessment characteristics are listed in Table 3.

**Table 3:** The factor structure assessment characteristics

Statistic	χ^2^	df	p	CFI	TLI	RMSEA	SRMR
Value for model fit with 5 subscales	3908	160	< 0.001	0.866	0.841	0.098	0.062

### PACIC score

The overall mean PACIC score was 2.44 (± 0.04). Subscale mean scores varied with the highest value of 3.26 (± 0.04) for delivery system design/decision support and the lowest value of 1.99 (± 0.04) for follow-up/coordination (Table 4).

**Table 4:** Total PACIC score, five subscales, and item scores among respondents with type 2 diabetes (*N* = 2,696**)

		n		Mean (SD)	Median	Missing (%)	Floor (%)	Ceiling (%)
Total score			2,696^*^	2.44 (0.02) *				
**Patient activation scale**			2,696^*^	2.66 (0.03) *				
	Item 1		2,680	3.21 (0.03)	4	4.6	27.6	33.1
	Item 2		2,647	2.19 (0.03)	1	5.8	52.4	13.1
	Item 3		2,663	2.69 (0.03)	2	5.2	40.7	23.3
**Delivery system** design/decision support scale			2,696^*^	3.26 (0.02) *				
	Item 4		2,670	2.45 (0.03)	2	5.0	45.0	16.4
	Item 5		2,695	4.03 (0.03)	4	4.1	8.5	47.8
	Item 6		2,678	3.43 (0.03)	4	4.7	19.7	35.0
**Goal setting scale**			2,696^*^	2.29 (0.02) *				
	Item 7		2,673	2.87 (0.03)	3	4.9	32.2	22.4
	Item 8		2,672	2.63 (0.03)	3	4.9	37.6	16.1
	Item 9		2,661	1.83 (0.03)	1	5.3	69.9	12.0
	Item 10		2,671	1.61 (0.02)	1	4.9	74.4	5.8
	Item 11		2,695	2.63 (0.03)	3	4.1	36.2	15.6
**Problem solving/contextual counselling scale**			2,696^*^	2.40 (0.02) *				
	Item 12		2,667	3.16 (0.03)	4	5.1	27.4	28.1
	Item 13		2,671	2.05 (0.03)	1	4.9	59.0	11.0
	Item 14		2,663	2.17 (0.03)	1	5.2	54.0	12.6
	Item 15		2,677	2.33 (0.03)	2	4.7	48.1	14.3
**Follow-up/ coordination scale**			2,696^*^	1.99 (0.02) *				
	Item 16		2,682	1.62 (0.02)	1	4.6	74.5	6.9
	Item 17		2,675	1.57 (0.02)	1	4.8	74.0	4.1
	Item 18		2,673	1.78 (0.03)	1	4.9	66.5	6.6
	Item 19		2,677	2.82 (0.03)	3	4.7	37.8	25.4
	Item 20		2,671	2.28 (0.03)	1	4.9	53.9	16.5

* Based on imputed data

** Out of 2,810 respondents, 114 respondents were excluded due to missing completed items, resulting in 2,696 respondents, who completed more than ten items

The effect parameters for variables with a statistically significant effect on total and subscale scores are shown in Tables 5 and 6.

**Table 5:** Significant effect parameter estimates for total PACIC score in linear regression models

	Estimate (SD)	*P* value
Intercept	0.20 (0.56)	0.71
Age	0.042 (0.017)	0.01
Age squared	-3.8e-4 (1.3e-4)	0.002
Annual control visits	0.058 (0.024)	0.02
Emergency department visits	-0.083 (0.030)	0.005
Primary diabetes care location		
General practitioner	0.64 (0.09)	< 0.001
Outpatient clinic	0.93 (0.10)	< 0.001
Private specialist	0.65 (0.27)	0.02
Rehabilitation program location		
Patient education		
Municipality	0.15 (0.07)	0.04
General practitioner	0.62 (0.05)	< 0.001
Outpatient clinic	0.34 (0.05)	< 0.001
Physical training		
Municipality	0.18 (0.06)	0.004
General practitioner	0.24 (0.08)	0.004
Outpatient clinic	0.40 (0.08)	< 0.001
Dietary advice		
Municipality	0.50 (0.07)	< 0.001
General practitioner	0.36 (0.05)	< 0.001
Outpatient clinic	0.31 (0.05)	< 0.001

**Table 6:** Effect parameters for PACIC subscale scores in linear regression models (*N* = 2,696*)

	Patient activation		Delivery system design/decision support		Goal setting		Problem solving/ contextual counselling		Follow-up/ coordination	
	Estimate	*P* value	Estimate	*P* value	Estimate	*P* value	Estimate	*P* value	Estimate	*P* value
Intercept	-0.13	0.88	-0.17	0.81	1.52	< 0.001	1.37	< 0.001	-0.17	0.77
Age	0.065	0.007	0.068	< 0.001					0.048	0.006
Age squared	-5.7e-4	0.002	-5.5e-4	< 0.001	-8.2e-05	< 0.001	7.5e-05	< 0.001	-4.1e-4	0.002
Men	-0.12	0.02								
Living alone	-0.12	0.02								
Planning area		0.04		0.008						
2	-0.069	0.32	-0.11	0.07						
3	-0.19	0.02	-0.24	< 0.001						
4	-0.12	0.09	-0.13	0.02						
5	-0.33	0.02	-0.13	0.28						
Education						0.009				0.009
Short					-0.066	0.12			-0.10	0.009
Medium					-0.079	0.35			-0.077	0.33
Long					-0.24	< 0.001			-0.21	< 0.001
Comorbidities										
Stroke	-0.23	0.04	-0.26	0.005						
Heart disease			-0.13	0.02						
Multimorbidity					0.22	0.04	0.29	0.03		
Utilization										
Annual control visits	0.09	0.01								
Planned hospitalization			-0.013	0.04						
Emergency department visits							-0.10	0.01	-0.09	0.002
GP visits									0.0045	0.009
Unplanned hospitalizations					0.062	0.007				
Out patient visits			-0.0048	0.04						
Readmissions	-0.14	.009								
Primary location of diabetes care										
General practice	0.66	< 0.001	1.13	< 0.001	0.48	< 0.001	0.58	< 0.001	0.45	< 0.001
Out patient Clinic	1.06	< 0.001	1.30	< 0.001	0.83	< 0.001	0.86	< 0.001	0.67	< 0.001
Private specialist			0.77	0.02	0.72	< 0.001	0.63	0.05		
Location of rehabilitation program										
Education										
Municipality					0.23	0.005			0.19	0.01
General practice	0.61	< 0.001	0.59	< 0.001	0.62	< 0.001	0.80	< 0.001	0.50	< 0.001
Out patient clinic	0.43	< 0.001	0.34	<0.001	0.37	< 0.001	0.35	< 0.001	0.28	< 0.001
Physical training										
Municipality	0.32	< 0.001	0.17	0.02	0.17	0.01	0.21	0.008	0.15	0.02
General practice	0.34	0.004	0.24	0.02	0.31	< 0.001	0.24	0.03		
Out patient clinic	0.42	< 0.001	0.43	< 0.001	0.43	< 0.001	0.50	< 0.001	0.29	< 0.001
Dietary advice										
Municipality	0.42	< 0.001	0.56	< 0.001	0.57	< 0.001	0.51	< 0.001	0.53	< 0.001
General practice	0.34	< 0.001	0.46	< 0.001	0.38	< 0.001	0.44	< 0.001	0.26	< 0.001
Out patient clinic	0.37	< 0.001	0.32	< 0.001	0.27	< 0.001	0.31	< 0.001	0.30	< 0.001

* Out of 2,810 respondents, 114 respondents were excluded due to missing completed items, resulting in 2,696 respondents, who completed more than ten items

### Age and sex

**Fig. 1:**
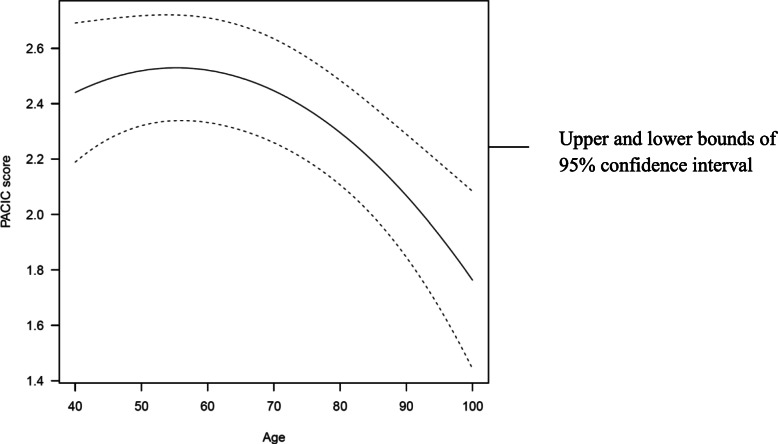
The effect of age on total PACIC score after adjusting for all other covariates

Total PACIC scores and subscale scores were inversely correlated with age and declined with increasing age from the age of approximately 70 years (Fig. 1). The effect was nonlinear for the total score and all subscales. A similar pattern was found for the five subscales.

Sex did not affect the total PACIC score. Men tended to have a lower score than women on the patient activation subscale, although the p-value of 0.02 did not indicate strong statistical significance.

### Sociodemographic variables

Educational level, occupation, and socioeconomic area did not affect the total PACIC score (Table 6). Educational level affected subscale scores to a significantly negative degree for goal setting and follow-up/coordination (p = 0.009 for both). For both subscales, patients with the most education scored significantly lower than those with less education (Table 5). Occupation did not affect any subscales. Socioeconomic area affected patient activation (p = 0.04) and delivery system design/decision support (p = 0.008). For the latter, respondents living in planning area three had markedly lower scores than respondents living in the other areas; compared to a composite of all other areas, the estimated mean subscale score for respondents in planning area three was 0.16 lower (*p* = 0.005).

### Diabetes treatment and rehabilitation

Respondents whose primary setting for diabetes care was a hospital outpatient clinic scored significantly and consistently higher for the total PACIC score on all subscales than respondents who did not attend a primary setting for care (Tables 4 and 5). Only twelve respondents reported a private specialist as the primary place for their diabetes care; their total PACIC scores were significantly higher than respondents who did not report a primary setting for diabetes care, but the effect was inconsistent across the subscales. Patients who reported receiving diabetes care at the hospital clinic had total PACIC scores that were an estimated 0.29 higher than patients who received diabetes care at their GP or a private specialist. However, the difference is borderline significant (*p* = 0.05). Patients who received diabetes care primarily at their GP or a specialist had an estimated 0.64 higher total PACIC scores than respondents who replied no primary setting for diabetes care or indicated “other” (*p* < 0.0001).

Participation in rehabilitation programs was associated with increased total and subscale scores except for smoking cessation (Tables 4 and 5). However, the effect varied according to the program location. Diabetes education with a GP showed the highest effect on the total PACIC score of all rehabilitation types, with an estimated rise in the total PACIC score of 0.62 (± 0.10). The effect on the total PACIC score from physical training was highest in the outpatient clinic, while dietary counselling yielded the highest increase in total PACIC score when it occurred in the municipality (Table 4).

### Contact with the healthcare system

The total PACIC score increased with the number of annual GP visits (*p* = 0.02) and decreased with the number of emergency department visits (*p* = 0.005, Tables 3 and 4). No consistent pattern occurred for the subscales. The patient activation subscale score decreased with the number of readmissions (*p* = 0.009) and for patients with a stroke diagnosis (*p* = 0.04) but increased with the number of annual GP visits (*p* = 0.01). Delivery system design/decision support subscale scores were lower for patients with a stroke diagnosis (p = 0.005) or a chronic heart condition (*p* = 0.02) and decreased with the number of outpatient contacts (*p* = 0.04) and planned hospitalisations (*p* = 0.04). Goal setting subscale scores decreased with the number of acute hospitalisations (*p* = 0.007) and increased with multimorbidity status (*p* = 0.04). Problem-solving/contextual counselling subscale scores decreased with the number of emergency department visits (*p* = 0.01) and increased with multimorbidity status (*p* = 0.04). Follow-up/coordination subscale scores increased with the number of GP visits (*p* = 0.009) and decreased as emergency department visits increased.

## Discussion

The study showed that PACIC is reliable for assessing the quality of care that type 2 diabetes patients receive in Denmark. Central factors important to the quality of perceived care are having a hospital outpatient clinic as the primary organization for diabetes care, participating in a rehabilitation program, and having higher number of visits to the GP and outpatient visits. Compared to other healthcare systems, the low total PACIC score on 2.44 suggests a potentially lower quality of diabetes care in Denmark from the patients’ perspective. The validation of the PACIC scale in a Danish context showed an acceptable ceiling effect and inter-item correlation, but floor effects persist for several items.

The study showed that PACIC is reliable for assessing the quality of provided care for type 2 diabetes in a Danish setting. In relation to the five subscales, we identified several factors important to the quality of care (Table 6). Patients perceived that they received better care if they were younger, were women, received annual control visits with their GP, and had primary location of diabetes in an outpatient clinic. Patients experiencing planned hospitalisations, emergency department visits and readmissions perceived that the quality of care was lower. The results provided insight into variations in patients’ perception of provided care and can be used for targeting initiatives towards improved care.

### Study population

The sex distribution in our study population is consistent with earlier reports that men represent a higher proportion of the population with type 2 diabetes, with the standardised proportion ratio ranging from 1.1 to 1.4 in 1999–2011 [16]. The comparable ratio in our study population was 1.4, which differed significantly from 1.0 (p < 0.0001). The study populations mean age of 67.2 (40–97) years is slightly older than the mean age of 64 years in the Danish National Diabetes Register [17]. The proportion of individuals with only ten years of primary school (31.7 %) [18] is slightly different, yet comparable, to another Danish diabetes study population, in which 28 % had no more than 11 years of education [19] The comparable levels of education provides acceptable representativeness with respect to education.

### Quality of diabetes care and factors important to the quality of care

The lower total PACIC scores in all five subscales from patients receiving care primarily from a private specialist, compared to those receiving it at their GP or at the hospital outpatient clinic, may result from patients perceiving outpatient clinics to provide a higher level of specialist care. Also, patients with severe complications primarily receive hospital care and thus may receive more attention to their health status, which may result in a higher total PACIC score.

Participation in a rehabilitation program was, in general, associated with higher both total and subscale PACIC scores. The organisations receiving the highest mean total PACIC scores varied with the type of diabetes rehabilitation provided. GP-generated patient education scored highest, 0.47 higher than patient education in the municipality. Patients receiving physical training in the hospital scored 0.22 higher than patients training in the municipality. Dietician advice in the municipality scored 0.14 higher than in the GP setting. The varying total scores for sites and types of rehabilitation may point to opportunities for inter-organisational learning and improvement.

The PACIC score decreased significantly with an increasing number of emergency department visits, which has not been previously reported. Experiencing an acute event in and of itself may cause a negative perception of the healthcare system. An increasing number of readmissions is followed by a decreasing mean score for the patient activation subscale and might be related to respondents interpreting readmissions as low-quality care.

Longer education negatively impacted the mean score for the goal setting and subscales, possibly due to well-educated patients not perceiving goal setting as relevant. The mean score for the delivery system design/decision support subscale was significantly lower for respondents living in planning area three, but with no differences between mean subscale scores for patients living in the other planning areas after covariate adjustments. This may be explained by the characteristics of planning area three, which includes some of the most affluent communities. The healthcare utilisation pattern in area three differs from the other areas in the region; there is greater use of specialists in private practice and less use of outpatient visits and bed days. The impact of age on the total PACIC score was nonlinear, with no significant effect up to 60–70 years, where a steep decline occurred, and the estimated score dropped to approximately 2.2 at the age of 85 years (Fig. 1). To our knowledge, this effect has not previously been reported [7, 8]. Potential explanations include the possibility of treatment being perceived as less effective and failing to meet expectations due to age-related increases in disease severity and comorbidity. Also, as patients develop additional age-related conditions, they encounter more healthcare professionals. All subscales except follow-up/coordination were significantly related to age.

### Assessment of PACIC questionnaire – imputation ceiling and floor effect and CFI and TLI estimates

With an increase in the study population of 10.6 % after imputation, the relevance of imputation is debatable. However, analyses comparing PACIC scores as a whole and stratified by sociodemographic variables revealed a consistent tendency towards lower PACIC scores for respondents with imputed values, compared to respondents completing all items. This introduces an upward bias in the population consisting of respondents answering all twenty items. A potential explanation is that the number of items answered decreased with age, which was also associated with lower total PACIC scores beginning around age 70 (Fig. 1). However, other characteristics of partial non-respondents may affect the PACIC scores. Thus, using imputed values was considered an appropriate improvement in the representativeness of the dataset.

The levels of floor and ceiling effects call for further investigation on specific items, particularly for those with high floor effects. The inter-item correlation is acceptable according to a lower limit of 0.15 [20], just as the item-rest correlation for fifteen of twenty items was higher than the maximum correlations with other scales, indicating item convergence within subscales. The values of Cronbach’s alpha met acceptability criteria according to McDowell [21] and Sitzia [22]. Factor loadings from the confirmatory factor analysis were high. The model χ^2^ test was strongly significant (Table 5). While undesirable, it is commonly seen in studies with large sample sizes (as ours and lower), even if the model is appropriate [23]. CFI and TLI estimates were slightly below the lower limit of 0.90 for acceptable fits [24], while RMSEA and SRMR appeared below upper the upper limits, 0.10 and 0.08, respectively, for acceptable fits [24, 25]. The evaluation of the PACIC questionnaire in this setting is thus consistent with a similar evaluation in Maindal [8] and surpasses it with respect to inter-item and item-rest correlation. In general, it is rather difficult to fit CFA models to PACIC evaluations. None of the studies we have encountered in our literature research fulfilled the criteria for a good fit i.e. non-significant χ^2^, CFI, TLI > 0.95, and RMSEA < 0.05 (e.g. [1, 8, 26, 27].) In summary, while challenges persist in the form of floor effects for several items, the data characteristics indicate that PACIC is a relevant tool for assessing patient-centred chronic care in Danish settings.

### Limitations

The statistical significance should be interpreted cautiously due to many investigated effects; the significance levels of *p* < 0.01 possibly arose from numerous comparisons. However, some of these findings with significant but high *p*-values are supported by patterns across the subscales, e.g. the effect of physical training in the municipality. Statistical significances with *p* < 0.001 are not questionable as this corresponds to a full Bonferroni correction. In the present study, there is a low response rate of 54.1 %. In our analyses of non-responders vs. responders we found that the non-responder group present a picture of people who, on average, are more socio-economically deprived in their lives than the responders and therefore more unlikely to complete the questionnaire. With the high rate of non-responders this may cause the results to be biased towards a more socio-economically well-functioning population.

## Conclusions

In conclusion, the PACIC questionnaire has potential as a tool measuring regular assessment of patient’s perspectives on the quality of care in a diabetes population. The PACIC questionnaire can document areas that need to be improved in the three healthcare organizations; hospitals, communities, and general practice offices. Factors important to a high quality of perceived care are age lower than 6o years, being a woman, having a hospital outpatient clinic as the primary organization. Also participating in a rehabilitation program are associated with perceived quality of care. Results indicate that PACIC is a relevant tool for assessing patient-centred chronic care in a Danish setting. Although, the validation of the PACIC scale in a Danish context showed an acceptable ceiling effect and inter-item correlation, but floor effects persist for several items.

## Supplementary Information

**Additional file 1: Supplementary figure S1.** The twenty-item Patient Assessment of Chronic Illness Care (PACIC) questionnaire

## Data Availability

The data used to support the findings of this study are available from the corresponding author upon request.

## Abbreviations

PACIC: The Patient Assessment of Chronic Illness Care.
CCM: The Chronic Care Model
GP: General practitioner
EM: The expectation-maximisation
RMSEA: The Root Mean Square Error of Approximation
SRMR: The Standardized Root Mean Square Residual
TLI: The Tucker-Lewis Index
CFI: The Comparative Fit Index
